# YOLO-S: A Lightweight and Accurate YOLO-like Network for Small Target Selection in Aerial Imagery

**DOI:** 10.3390/s23041865

**Published:** 2023-02-07

**Authors:** Alessandro Betti, Mauro Tucci

**Affiliations:** 1 FlySight srl, via A. Lampredi 45, 57121 Livorno, Italy; 2 Department of Energy, Systems, Territory and Construction Engineering, University of Pisa, 56122 Pisa, Italy

**Keywords:** aerial imagery, convolutional neural network, vehicle detection, feature fusion, reshape pass-through layer, computer vision

## Abstract

Small target detection is still a challenging task, especially when looking at fast and accurate solutions for mobile or edge applications. In this work, we present YOLO-S, a simple, fast, and efficient network. It exploits a small feature extractor, as well as skip connection, via both bypass and concatenation, and a reshape-passthrough layer to promote feature reuse across network and combine low-level positional information with more meaningful high-level information. Performances are evaluated on AIRES, a novel dataset acquired in Europe, and VEDAI, benchmarking the proposed YOLO-S architecture with four baselines. We also demonstrate that a transitional learning task over a combined dataset based on DOTAv2 and VEDAI can enhance the overall accuracy with respect to more general features transferred from COCO data. YOLO-S is from 25% to 50% faster than YOLOv3 and only 15–25% slower than Tiny-YOLOv3, outperforming also YOLOv3 by a 15% in terms of accuracy (mAP) on the VEDAI dataset. Simulations on SARD dataset also prove its suitability for search and rescue operations. In addition, YOLO-S has roughly 90% of Tiny-YOLOv3’s parameters and one half FLOPs of YOLOv3, making possible the deployment for low-power industrial applications.

## 1. Introduction

Small target detection in aerial imagery has become nowadays a hot research topic for several applications [1,2,3,4]. Indeed, the recent advent of data-enabling technology such as unmanned aerial vehicles (UAVs) represents a cost-effective solution for a broad customer base, satisfying a wide and almost limitless range of user needs depending on the camera axis, altitude of the craft and type of film used. Furthermore, the growing availability of publicly available vehicles data from either satellite or UAV-carried sensors [5,6,7] has pushed forward the research in the field. Nevertheless, the low resolution of the vehicles within the images, the poor distinctive features of tiny targets, the variability in vehicle type, size and color, as well as the presence of cluttered background or disturbing atmospheric agents, still represent a challenge for a satisfactory vehicle detection rate of Convolutional Neural Networks (CNNs). Moreover, the occurrence of confusing objects, such as containers, buildings, or road marks, may enhance the likelihood of false alarms. In addition, a reasonable trade-off between accuracy and latency time is necessary. Popular object detectors are memory hungry and can be executed generally only in centralized high-performance platforms. In particular, double-stage detectors [8,9,10] are not suitable for real-time detection, whereas single-stage detectors [11,12,13,14] provide real-time performances only on powerful resources. None of them is also adequately tailored for small target detection. In addition, many industrial applications require the deployment of CNNs locally on edge devices close to the data source because of cheaper and faster data processing, unreliable data exchange with a remote server, or security and privacy issues. However, such devices are usually characterized by limited hardware resources in terms of performance, cost and energy, and do not include GPUs. Hence, fast and lightweight CNNs are mandatory, while maintaining satisfactory accuracy even on small targets. Tiny-YOLOv3 [15] does not guarantee adequate performances due to the poor features extracted by its backbone and the coarseness of its output scales.

Alternative solutions are therefore now slowly emerging to detect small vehicles more precisely and quickly. Çintaş et al. [16] and Saribas et al. [17] combine Tiny-YOLOv3 for object detection from UAV (Unmanned Aerial Vehicle) with Kernelized Correlation Filter (KCF) for image tracking, outperforming in [16] Tiny-YOLOv3 by 18% in terms of accuracy on a newly presented dataset and improving FPS (frames per second) by running the YOLO stage once over 30 consecutive images when analyzing a video. The interesting model UAV-YOLO is presented in [18], which is based on YOLOv3, and where the Resblock in darknet is first optimized by concatenating two ResNet units that have the same width and height. Then, the entire darknet structure is improved by increasing convolution operation at an early layer to enrich spatial information. The results improve YOLOv3 in terms of accuracy, but the size of the model is not as small as Tiny-YOLOv3.

An interesting model, EfficientDet, is proposed by Tan et al. in [19] for efficient object detection, where the authors propose two key optimizations: a weighted bi-directional feature pyramid network (BiFPN), and a compound scaling method that uniformly scales the resolution, depth, and width for all backbone, feature network, and box/class prediction networks at the same time. As a result, EfficientDet has roughly the same accuracy with respect to YOLOv3, being 28× faster and having only 6% of the parameters. EfficientDet is not particularly optimized for small object detection. As a comparison, our model YOLO-S is 2× faster and uses the 12% of YOLOv3 parameters, but significantly outperforms YOLOv3 for small object detection; for example, mAP is improved by 15% on the VEDAI dataset.

In [20], a modified YOLOv5 network is proposed that improves YOLOv5 mAP by 1%, and YOLOv3 by 2% on drone images. He et al. [21] propose TF-YOLO, which keeps the same backbone of Tiny-YOLOv3 but introduces one more output scale as in YOLOv3, and lateral connection among multiple layers. By also estimating more robust anchors by means of k-means clustering based on Jake’s distance, on the NWPU VHR-10 dataset [22,23], TF-YOLO outperforms Tiny-YOLOv3 with a mean Average Precision (mAP) almost 6% higher and a similar speed of roughly 24 Frames Per Second (FPS). On a subset of the VOC2007 dataset [24], TF-YOLO obtains a mAP (mean Average Precision) of 31.5% and a speed of 11.1 FPS (frames per second), resulting in being 24.4% less accurate and almost 30.8% faster than YOLOv3, and 4.3% more accurate and 10% slower than Tiny-YOLOv3. Manual design of network architectures may include not vital layers. Zhang et al. [25] conceive therefore an automated iterative procedure of incremental model pruning and apply it to YOLOv3-SPP3, which is a modified version of YOLOv3 with Spatial Pyramid Pooling (SPP) [26] added on top of the three heads in order to extract superior multi-scale features based on four main steps: (i) apply channel-wise sparsity to identify the less important channels in each convolutional layers, (ii) remove the useless channels based on predefined thresholds, (iii) then fine-tune the model, and (iv) finally evaluate the pruned model to determine the suitability for deployment; otherwise, restart from (i). The lightest pruned model SlimYOLOv3-SPP3-95 obtains a mAP on images 416 × 416 of the VisDrone DET2018 dataset [27] with an 18% relative drop compared to YOLOv3-SPP3, but resulting in being 80% faster and occupying just 8% of YOLOv3-SPP3’s volume, which corresponds to 59% of Tiny-YOLOv3’s volume. Ju et al. [3] introduce a simple, fast and accurate network composed by 31 convolutional layers, one reshape–passthrough layer and a single output scale. To expand quickly the receptive field and obtain more contextual information around targets avoiding any information loss, they implement dilated convolution instead of downsampling based on strided convolution. Furthermore, they employ reshape–passthrough layers and feature fusion to merge features from earlier layers with those of deeper layers and improve the overall localization performances. On VEDAI (Vehicle Detection in Aerial Imagery), the network achieves a mAP of 47.8%, i.e., 8.0% less accurate than YOLOv3, while 30.0% more accurate than Tiny-YOLOv3. On an Intel i7-5930k processor and TITAN X GPU, it can process almost 75 FPS, resulting in being about 5 times quicker than YOLOv3 and nearly as fast as Tiny-YOLOv3.

In [28], the authors observe that, assuming images acquired from a camera besides the road, rather than from UAV, the background does not change significantly. Hence, in this constrained environment, batch normalization (BN) layers after convolution operations are not necessary. As a consequence, starting from Tiny-YOLOv3, authors trim manually and incrementally BN layers as well as whole convolutional layers which do not precede max pooling layers. The lightest trimmed network they obtain is tested over the BIT-vehicle dataset, where targets may extend up to a few tens of percent of the image size, achieving a mAP very close to YOLOv3 and with a speed a bit higher than Tiny-YOLOv3.

Other research works trade instead accuracy for speed. In [29], a fourth output scale 104×104 is added to YOLOv3 in order to decrease the receptive field, obtaining a 3% mAP improvement on DOTA (Dataset for Object deTection in aerial images), despite of a slower inference. Since YOLOv3 mainly detects small targets at the scale 52 × 52, Ref. [30] proposes YOLO-E based on two outputs 52 × 52 and 104 × 104 and implements a two-way residual submodule in order to make the network less deep. They also improve the sensitivity to target position by replacing the Intersection over Union (IoU) metric with GIoU [31] and adding a new term 1—GIoU to the YOLOv3 loss function. On VEDAI, it obtains a mAP of 91.2%, almost a fifth more accurate and 6.7% slower than YOLOv3. In [32], a cascaded detector is presented based on the VGG16 architecture [33], outperforming Faster R-CNN on VEDAI and Munich datasets at the price of a 20–30% slower inference. In addition, low-resolution aerial imagery worsens the extraction of meaningful features from vehicles due to their appearance ambiguity and similarities with the context. In [34], it is proven that a joint-learning of two super-resolution and detection networks conducts to more meaningful targets and a higher perceptual quality in the super-resolved image, which in turn leads to an enhanced accuracy in the detection task and performances on low-resolution aerial imagery close to the existing state-of-the-art methods fed with the corresponding high-resolution images. To handle this problem, Ref. [34] proposes a Joint Super-Resolution and Vehicle Detection Network (Joint-SRVDNet) that leverages complementary information of the two inter-related super-resolution and detection tasks. Joint-SRVDNet is composed by two main modules: a multi-scale MsGAN for image super-resolution with a 4× upsampling factor and YOLOv3 for vehicles detection. Specifically, authors demonstrate that a joint-learning of the two networks allows for obtaining more meaningful targets and a higher perceptual quality in the super-resolved image, which in turn lead to an enhanced accuracy in the detection task and performances on low-resolution aerial imagery close to the existing state-of-the-art methods fed with the corresponding high-resolution aerial images.

Hence, fast and accurate small vehicle detection remains nowadays a debated issue that encourages further research in this area. In particular, as far as detection of tiny targets from aerial images is concerned, Tiny-YOLOv3 does not guarantee an adequate accuracy [3]. In this work, we present a novel YOLO-like network, namely YOLO-S or YOLO-*small*, and we evaluate its performances against some well-known baseline detectors. More specifically, the contributions of this paper are the following:

1. We design YOLO-S, a small and fast network with a single fine-grained output scale and exploiting residual connection and feature fusion of 4×, 8× and 16× down-sampled feature maps via upsampling and reshape–passthrough layers in order to strengthen feature propagation and reuse, and improve target position accordingly.
2. We design also YOLO-L, or YOLO-*large*, a baseline CNN detecting at three different resolution levels corresponding to 4×, 8× and 16× down-scaled layers, but more focused on accuracy and suitable only for offline data processing due to the FPS close to YOLOv3.
3. We prepared two different vehicle datasets for experiments: VEDAI and AIRES, a new dataset for cAr detectIon fRom hElicopter imageS. In addition, we make experiments on SARD (SeArch and Rescue image Dataset) [4] to verify how YOLO-S generalizes to the context of search and rescue (SAR);
4. We compare YOLO-S with four baselines: YOLOv3 [12], Tiny-YOLOv3, the model presented in [3] and YOLO-L. Experiments are performed by either applying a sliding window based inference or a detection on full-sized images;
5. We also propose a double-stage training procedure by first fine-tuning on a dataset composed of DOTA and VEDAI, and then by training on the specific vehicle dataset of interest. In fact, usually a domain knowledge transfer is realized by fine-tuning a learning model starting from pre-trained weights on COCO or ImageNet [35]. However, since large publicly available datasets do not include small vehicles from aerial images, the basic single-stage trained model may be less efficient due to a domain gap and can under-perform with respect to the proposed double-stage training.

Results highlight that YOLO-S is really a cost-effective solution for practical applications, outperforming the other baselines in terms of accuracy, and resulting in being up to 50% faster than YOLOv3 and competitive with Tiny-YOLOv3 and [3]. YOLO-based methods are selected as the baseline for two reasons. A first reason is that the proposed models YOLO-S/L are custom architectures that exploit the same working philosophy of YOLOv3 and the same loss function, so it is reasonable to use YOLOv3 as a baseline. Secondly, even in the most recent versions of YOLO that are now appearing in the literature, such as [3,16,17,18,25,28], YOLOv3 is commonly used as a reference point, since, until recently, it was the single-stage architecture to beat having the best accuracy-time latency trade-off. The rest of the paper is organized as follows: Section 2 explores the adopted datasets and presents the methodology, Section 3 discusses the results, whereas conclusions are summarized in Section 4.

## 2. Materials and Methods

### 2.1. AIRES: The Proposed Vehicles Dataset

In this paper, we introduce AIRES (cAr detectIon fRom hElicopter imageS), a new vehicle database composed by aerial full high-definition (FHD) images, with a resolution of 1920 × 1080, streamed by a WESCAM MX-15 EO/IR imaging system placed in a multi-sensor 4-axis gyro-stabilized turret system and mounted at the front end of a manned police helicopter AW169. The helicopter flew at different altitudes from almost 300 m to 1000 m and different camera angles ranging from about 5° to 80°. The images were acquired from June to September 2019 in two different geographical areas: the region of Lombardia in the north of Italy and the city of Oslo in Norway. The dataset is composed by 1275 images annotated with the LabelImg software [36] and containing 15,247 annotated ground truth (GT) objects organized in eight classes: *Van*, *Truck*, *Car*, *Motorbike*, *Person*, *Other*, *Boat* and *Bus*. The statistics are summarized in Table 1: the majority class is *Car*, whereas the less populated ones are *Motorbike*, with 0.5% and *Other* with 0.8%, the latter including bulldozer and other ground moving vehicles employed in construction sites.

**Figure 1:**
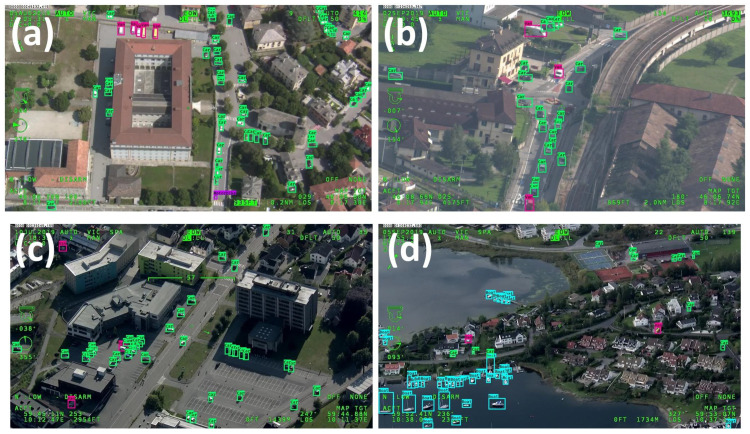
Some images of the AIRES dataset: (**a**,**b**) have been collected in Italy, whereas (**c**,**d**) in Norway. The vehicles are delimited by GT bounding boxes.

**Figure 2:**
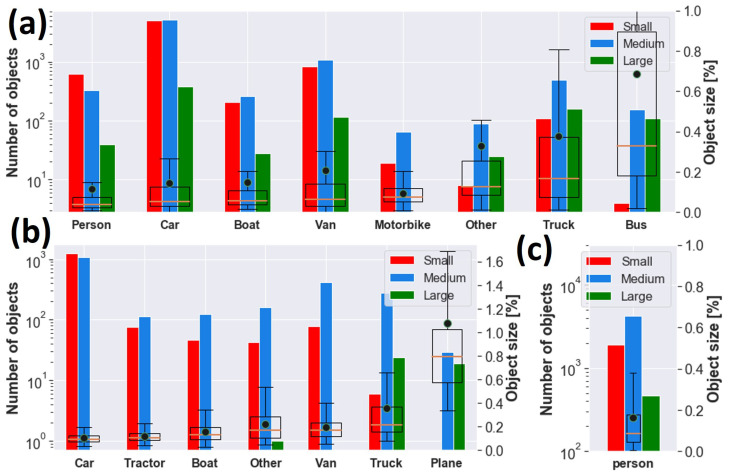
GT objects for (**a**) AIRES, (**b**) VEDAI and (**c**) SARD datasets. **Left**: histogram of small, medium and large objects grouped by class according to COCO convention [37]. **Right**: box plot with 25th, 50th and 75th percentiles of target area for each class as a percentage of the image size. The mean area is shown as a black circle.

**Figure 3:**
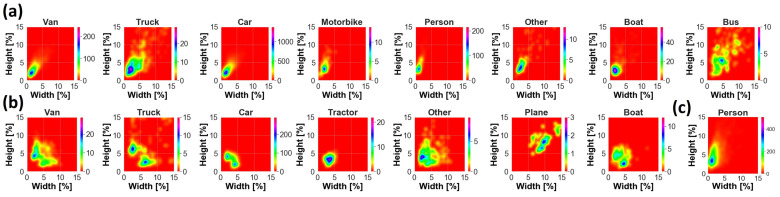
Two-dimensional density plot of GT objects in the plane (GT width, GT height) for (**a**) AIRES, (**b**) VEDAI and (**c**) SARD datasets. GT size is normalized by image size. Image shape is 1920 × 1080 for (**a**,**c**) and 1024 × 1024 for (**b**).

Data are characterized by variability of target dimension due to acquisition at different altitudes and multiple viewpoints, as well as by the presence of different backgrounds, such as urban or rural areas, lighting conditions and target occlusion, and blurring, as shown in Figure 1a. According to COCO’s protocol, objects can be categorized into *small*, *medium* and *large*. *Medium* targets are usually the most representative size for each class, as can be appreciated in Figure 2a. *Small* objects are widely present as well for *Car*, *Van* and *Person*. The largest target is *Bus*, with a median area of almost 0.33%, i.e., 83 × 83 pixels, whereas the smallest target is *Person*, with a median area of almost 0.04%, i.e., 29 × 29 pixels. Samples typically have a width and height smaller than a few percent, except mostly for classes *Bus*, *Truck* and *Other*, where a remarkable presence of objects of up to 10–15% occurs, as shown in Figure 3a.

### 2.2. DOTAv2 and VEDAI Datasets

We also used the publicly available vehicle datasets DOTAv2 [38] (Dataset for Object deTection in aerial images) and VEDAI [39] (Vehicle Detection in Aerial Imagery). VEDAI is a dataset of 1246 aerial images 1024 × 1024 cropped from satellite images of the Utah Automated Geographic Reference Center [40] and including 12 classes. To make VEDAI and AIRES consistent with each other, we merged *car* and *pickup* from VEDAI as one class *Car*, and *camping_car* and *van* as *Van*. In addition to the classes used in AIRES, we also exploited the categories *Tractor* and *Plane* present in VEDAI, as reported in Table 1. Globally, 3750 ground truths were used from VEDAI dataset, with *Car* representing roughly 62.2% of the overall statistics, while *Plane* has the lower presence of 1.3%. Medium sized objects are predominant for all categories, except *Car*, for which small and medium groups include more than one-thousand instances each, as shown in Figure 2b. Large objects statistics is instead under-represented and mainly available for *Plane* and *Truck*. The median size of the targets ranges from 0.09% for *Car*, with 31 × 31 pixels, up to roughly 0.22% and 0.80%, i.e., 48 × 48 and 92 × 92 pixels, for *Truck* and *Plane*, respectively, as shown in Figure 2b and Figure 3b. DOTAv2 is a large-scale dataset composed by satellite images collected by Google Earth, GF-2 and JL-1, as well as by aerial images provided by CycloMedia. It consists of eighteen categories and 2421 annotated training/validation images with variable size from a few Megapixels up to more than 100 Megapixels. We selected 314,903 targets grouped in *Small-Vehicle*, *Large-Vehicle*, *Ship* and *Plane*, where the first three classes have been renamed as *Car*, *Truck* and *Boat*, respectively.

### 2.3. The Search and Rescue Dataset (SARD) for Person Detection

We also used the SARD dataset [4] (SeArch and Rescue image Dataset), which is a collection of 1980 FHD aerial images and 6525 instances of class *Person*, as shown in Table 1, extracted from a 35-minute long video acquired from UAV and built for search and rescue operation (SAR) of people in distress or danger [41]. The frames show actors simulating standard positions, such as standing, sitting, walking, running and lying, as well as positions typical of exhausted or injured persons. Furthermore, they include backgrounds such as woodland edge, low and high grass, lake basin, quarries and macadam roads. The median area is less than 0.1% as shown in Figure 2c.

### 2.4. The Proposed Networks YOLO-L and YOLO-S

**Figure 4:**
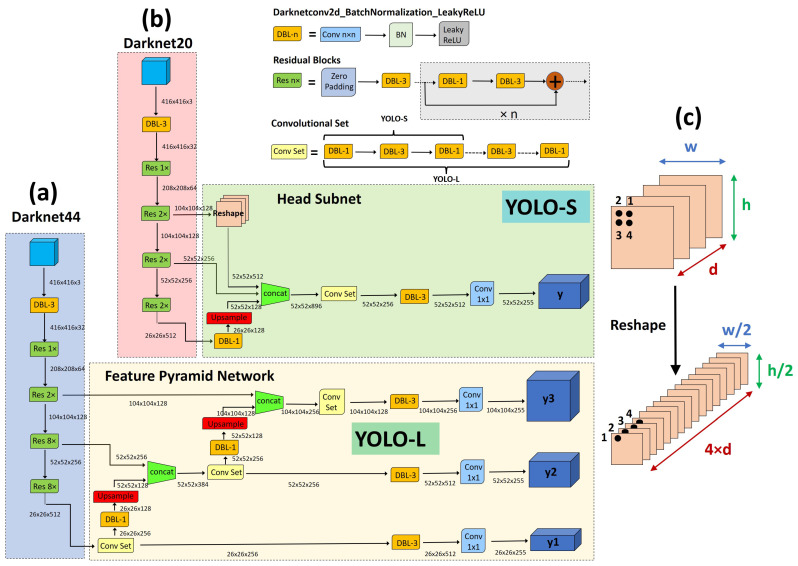
Overview of the proposed networks: (**a**) YOLO-L; (**b**) YOLO-S; (**c**) Reshape–Passthrough layer. In the figure, C=80 classes have been assumed (dataset COCO [37]).

In this work, we propose two novel YOLO-like architectures: YOLO-L and YOLO-S, whose architecture is depicted in Figure 4. Full details about the proposed CNNs are available in Table 2, where we also report the receptive field and cumulative stride for each layer. We assume an input image resized to the default size 416 × 416. YOLO-L, namely YOLO-*large*, is proposed mainly for benchmarking purposes due to the limited inference speed which makes it suitable only for offline processing on high-power hardware. YOLO-S, or YOLO-*small*, is instead our proposal for an efficient, lightweight and accurate network to be deployed on edge devices.

Further details are provided in Table 3, where the proposed networks are compared to other state-of-the-art detectors, in terms of number of parameters, volume, BFLOPs and architecture properties.

Receptive field $RF_{k}$ of layer *k* has been computed according to the formula:

(1) $$RF_{k}=RF_{k-1}+\left(f_{k}-1\right)\times CS_{k-1},$$

where

(2) $$CS_{k-1}=\prod_{i=1}^{k-1}s_{i}$$

is the cumulative stride of layer k−1, $f_{k}$ is the filter size of layer *k*, and $s_{i}$ is the stride of layer *i*, respectively.

The larger network YOLO-L, shown in Figure 4a, employs Darknet44 as backbone and a head subnet based on a Feature Pyramid Network (FPN) [42] to make detection at three different scales: 26 × 26, 52 × 52 and 104 × 104. The rationale behind this choice is the fact that high-resolution feature maps contain grid cells that cover smaller regions of the input space or, equivalently, have a small receptive field, and are therefore more suitable for detecting smaller objects. Hence, in order to make the network more focused on small targets, we decrease the receptive field of YOLO-L with respect to YOLOv3 by replacing the coarse-grained YOLOv3’s output scale 13 × 13 with a finer-grained 4× down-sampled feature map of size 104 × 104, as can be observed in Table 3. In addition, a smaller backbone is also used, i.e., Darknet44, removing the last four residual blocks of Darknet53 corresponding to 32× down-sampled layers, which were used in YOLOv3 to downscale the image down to 13 × 13. YOLO-L includes almost 23.848M of parameters, i.e., roughly 38.7% of YOLOv3’s weights, and requires 90.53 BFLOPs for each input image, as shown in Table 3.

YOLO-S, shown in Figure 4b, is instead a tiny and fast network that exploits the concepts of feature fusion and reshape–passthrough layer, depicted in Figure 4c, to combine precise location information of earlier fine-grained feature maps with meaningful semantic information from deeper feature maps having lower resolution. Basically, it is based on a Darknet20 backbone, replacing the max pooling layers of Tiny-YOLOv3 in the feature-extraction stage with strided convolutional layers and residual units to reduce information loss during downsampling and increase efficiently the receptive field. The lightweight backbone, composed by seven residual blocks, also allows for avoiding useless convolution operations for small-scale detected objects, which otherwise in a deeper architecture could lead to final features with a few pixels left after multiple down-sampling. In addition, YOLO-S employs a head subnet with one single output scale 52 × 52 and a smaller convolutional set of just 4 alternate convolutional layers 1 × 1 and 3 × 3, instead of 6 as in YOLO-L and YOLOv3, to speed up inference. This leads to a receptive field of the output as large as 101 × 101, sufficient to obtain meaningful contextual information around targets once source images are rescaled to the size expected by the network.

Finally, skip connection is implemented to extract more robust localization features by laterally connecting the eighth, thirteenth and nineteenth layers of the backbone, corresponding to 4×, 8× and 16× down-sampled feature maps, respectively. Since such feature maps exhibit different resolutions, upsampling is applied to the nineteenth layer, and reshaping to the eighth layer, to match every size to the shape 52 × 52 before concatenation. Overall, YOLO-S has a model volume that is shrunken by 87% with respect to YOLOv3 (the size of YOLO-S is only the 7.9% of YOLOv3) and contains almost 7.853M of trainable parameters, resulting even 10% lighter than Tiny-YOLOv3. In addition, it requires 34.59 BFLOPs, which is close to SlimYOLOv3-SPP3-50 [25] and almost one half of YOLOv3, as shown in Table 3. However, the proposed model YOLO-S outperforms YOLOv3 in terms of accuracy in the experiments reported in this work.

### 2.5. State-of-the-Art Detectors

We re-implemented the following state-of-the-art single-stage detectors for benchmarking: YOLOv3 [12], Tiny-YOLOv3 [15] and the model presented in [3]. YOLOv3 exploits Darknet53 and a head subnetwork based on a FPN with three output scales from 13 × 13 to 52 × 52 to detect both large and small targets. It also implements features fusion via a top-down pathway and lateral connection between feature maps of different resolution to obtain accurate location information.

Each output scale of the network can be represented by a grid where its resolution controls the size of the portion of the input image an output cell can see. In particular, each grid cell outputs an array with length B×(5+C) by solving a regression problem, where *B* is the number of bounding boxes a cell can predict, 5 stands for the number of bounding box attributes and the object confidence, and *C* is the number of classes. Pruning is finally applied to the detected items by means of Non Maximal Suppression (NMS) to preserve only the highest confidence ones.

Overall, YOLOv3 includes 61.603M of parameters, as shown in Table 3.

Tiny-YOLOv3 has instead 5.58 BFLOPs and almost 8.688M of parameters obtained by replacing Darknet53 with a backbone composed by 7 convolutional and 6 max pooling layers, as can be seen in Table 3. It makes detection at 13 × 13 and 26 × 26, thus resulting in being less efficient in identifying small targets. The network designed by [3] has instead an output scale equal to 52 × 52, which corresponds to a receptive field 133 × 133. The use of 1 × 1 convolution after each concatenation allows for obtaining less than one tenth parameters of Tiny-YOLOv3, as shown in Table 3.

### 2.6. The Experiments

In order to evaluate the performance of the proposed models and compare it with respect to the other models, we defined a set of experiments on AIRES, VEDAI and SARD datasets.

#### 2.6.1. Experiment 1 on AIRES Dataset

In this experiment, we evaluate the CNNs on AIRES by using a double-stage training and a sliding window approach during inference.

**Figure 5:**
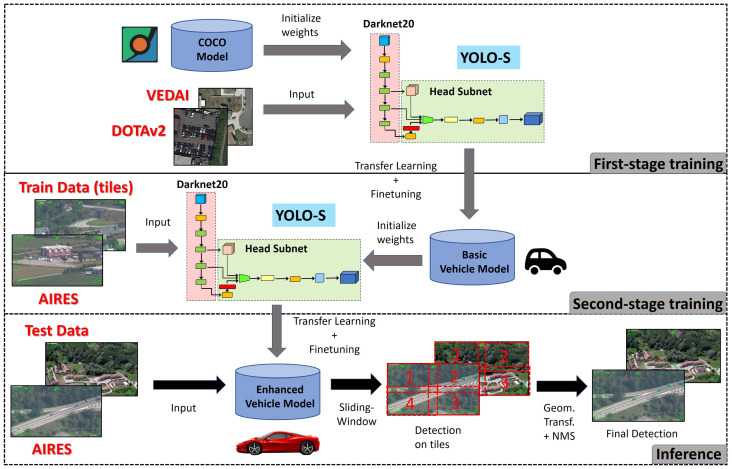
Workflow of the proposed vehicle detection approach for experiment 1 on AIRES dataset.

First, the dataset has been split randomly in training (70%) and test (30%) by implementing stratified sampling for each class (Table 1). Then, in order to enrich statistics available during model learning, we enabled standard data augmentation techniques including horizontal flipping, resizing, cropping and random distortion of brightness, contrast, saturation and hue. However, the over-fitting issue due to a lack of data cannot often be resolved effectively with only data augmentation methods, especially for minority classes. Hence, we adopted the so-called "transfer learning" technique in order to take advantage of knowledge achievable on publicly available databases. This is especially beneficial for accuracy: the more similar the base task, on which preliminary features are extracted, to the target task of interest, the higher the accuracy achievable [43]. The proposed training pipeline is illustrated in Figure 5.

Specifically, we implemented a double-stage training adding a transitional task between the source task based on COCO dataset and the target task on AIRES dataset: first training on DOTAv2+VEDAI (first training phase) and then on AIRES (second training phase). Pre-trained weights on COCO were used as a warm starting point to speed up training and improve accuracy.

To make the labels of VEDAI and AIRES consistent, during the first training phase, we used VEDAI statistics reported in Table 1, but excluding *Plane* and *Tractor*. We also exploited all the DOTAv2 instances of *Car*, *Truck* and *Boat* available. As the size of DOTAV2 images were not uniform, we cropped every DOTAv2 and VEDAI image in not-overlapping tiles having the minimum side not smaller than 416 pixels and retaining the original aspect ratio. Then, we executed first-stage training on the resulting 74,232 patches of joint dataset DOTAv2 + VEDAI by applying padding to make images square.

The second training phase on AIRES dataset was performed by starting from pre-trained weights on DOTAv2 + VEDAI. Training images were divided in patches in order to couple efficiently with the inference step. In particular, since image resizing from full-sized image to the network size reduces a *Bus* object, which is median-sized 83 pixels wide, as reported in Figure 2a, to just 18 pixels, we implemented a sliding window approach during inference assuming $N_{x}=N_{y}=2$ windows with an overlap of $o_{x}=o_{y}=50$ pixels, corresponding roughly to the third quartile of the population size. Specifically, the overlapping was set to cope with objects at the edge, and this choice is a trade-off between target localization accuracy and latency time. In fact, the latency time is proportional to the overlapping area due to the post-processing NMS, which is used to remove redundant detections over the same object in the overlapping regions after sliding and detection steps [44]. Hence, for consistency, we split each training image into 4 tiles of shape $\Delta_{x}\times \Delta_{y}=$ 985 × 565 pixel, where tile size was computed as

(3) $$\Delta_{i}=\left\lceil \frac{L_{i}+\left(N_{i}-1\right)\cdot o_{i}}{N_{i}}\right\rceil ,$$

where i=x,y and $L_{i}=W,H$ are the width and height of full size image, respectively. During both training stages, we assumed a two-stage pipeline by first training the last convolutional layers and then unfreezing the whole CNN.

#### 2.6.2. Experiment 2 on AIRES Dataset

In this experiment, we implement a single-stage training on AIRES dataset to check the performance drop. Pre-trained weights on COCO dataset were used.

#### 2.6.3. Experiment 1 on VEDAI Dataset, Double-Stage Sliding

In this experiment, we test the CNNs on VEDAI by using a double-stage training and a sliding window approach during inference.

VEDAI has been split preliminarily in training (70%) and test (30%), whereas DOTAv2 has been used entirely for training. Then, a preliminary training on DOTAv2 was performed starting from COCO weights, followed by a second training on VEDAI, as shown in Table 1. As in Section 2.6.1, inference was executed by sliding a window over the whole input image with $N_{x}=N_{y}=2$ and $o_{x}=o_{y}=50$ pixels. Hence, VEDAI training images were split into 4 tiles of size $\Delta_{x}\times \Delta_{y}=$ 537 × 537 pixel.

#### 2.6.4. Experiment 2 on VEDAI Dataset, Single-Stage Full-Sized

In this experiment, we implement a single-stage training on VEDAI and infer over the full-sized image. This method, which is the same applied in [3], aims to verify the robustness of the CNNs when a more computationally demanding sliding window strategy is not a feasible solution due to time constraints. We conducted a training on the whole VEDAI images directly based on pre-trained COCO weights.

#### 2.6.5. Experiment on SARD Dataset

Here, we implement a single-stage training on SARD dataset and infer over the full-sized image, as in experiment 2 on VEDAI, splitting data as 80–20%, as shown in Table 1.

#### 2.6.6. Evaluation Metrics

We introduce both the PASCAL VOC [45] and COCO [37] evaluation protocols. For each class, we compute the Average Precision (AP), defined as the area under the Precision–Recall curve for different Intersection over Union (IoU) between ground-truth and predicted boxes. Overall performance is computed by means of mAP, defined as

(4) $$\mathrm{mAP}=\frac{1}{n}\cdot \sum_{i=1}^{n}{\mathrm{AP}}_{i},$$

where the sum runs over the *n* classes. We also introduce the weighted Average Precision (wAP), where the *i*-th class is weighted according to its support $N_{i}$:

(5) $$\mathrm{wAP}=\sum_{i=1}^{n}\frac{N_{i}}{N}\cdot {\mathrm{AP}}_{i},$$

and $N=\sum_{i=1}^{n}N_{i}$ is the overall number of instances. Additionally, we compute COCO metrics AP_S_, AP_M_ and AP_L_ by applying AP@[0.5:0.05:0.95] and considering only ground truth objects of small, medium and large size, respectively [46]. Furthermore, as datasets exhibit class imbalance, we evaluate the micro-average recall $R_{\mathrm{ma}}$, precision $P_{\mathrm{ma}}$ and F1-score ${F1}_{\mathrm{ma}}$ by aggregating all contributions. Finally, we estimate the processing speed based on FPS.

## 3. Results and Discussion

All the networks have been implemented using the TensorFlow framework. Experiments were conducted using two processors: an Intel(R) Core(TM) i7-4770 CPU @ 3.40 GHz with 16 GB RAM and NVIDIA QUADRO RTX 5000 GPU used for both training and inference, and an Intel Xeon E5-2690 v3 with 56 GB RAM and NVIDIA Tesla K80 GPU exploited only for training. All algorithms adopt the same objective function based on YOLOv3, which has been minimized during training according to the stochastic Adam optimizer. A step-wise learning rate has been employed, starting from $10^{-3}$ and decreasing by a factor 10 after every 10 epochs without any loss improvement. Batch size was fixed to 32 during transfer learning and 8 during fine-tuning. The early stopping method was also used to avoid overfitting on training data by randomly leaving out 10% of training instances for validation purpose. The network by [3], as well as YOLO-L and YOLO-S were preliminarily trained from scratch on the COCO dataset, whereas pre-trained YOLOV3 and Tiny-YOLOv3 on COCO were exploited [15]. Then, the models were fine-tuned on the considered datasets for up to 1000 epochs.

We estimated anchor priors based on an enhanced k-means clustering by replacing the Euclidean distance with the IoU metric to avoid an error biased towards large bounding boxes [21]. Three anchors have been assumed for each output scale of YOLOv3, Tiny-YOLOv3 and YOLO-L and six anchors for [3] and YOLO-S. All results, but COCO metrics, correspond to an intermediate IoU equal to 0.5.

### 3.1. Experiments on AIRES Dataset

#### 3.1.1. Quantitative Comparison with State-of-the-Art Detectors

It may be seen from Table 4 that the proposed CNNs outperform all the other detectors in experiment 1, with a mAP of 46.7% for YOLO-S, 43.4% for YOLO-L and less than 14% for Tiny-YOLOv3. In general, the larger the statistics available, the higher the AP achievable, with remarkable values for Car and modest for Motorbike and Other. YOLO-S is also extremely robust, ranking first or second for almost every vehicle category. AP for class Car for YOLO-S is as much as 10.9% better than YOLOv3, and 12.2% better than [3]. This leads to a wAP for YOLO-S more than 10% higher than YOLOv3 and [3].

Furthermore, our CNNs show the best results for all object sizes, obtaining an AP ranging from 11.9% for small objects to 29.3% for large objects. Our networks are also better when looking at micro-average accuracy: YOLO-L has the best trade-off between true positives and false positives, with an F1_ma_ of 65.6%. Nevertheless, YOLO-S is very competitive with the best R_ma_ of 62.0%. YOLOv3 is instead not adequately tailored for small targets, as proven by the F1_ma_ degraded by 7.3% with respect to YOLO-L. It is also confirmed that [3] can almost catch up with performances of YOLOv3. On the contrary, features extracted by Tiny-YOLOv3 are very poor, leading to a F1_ma_ roughly one half of the proposed CNNs. Tiny-YOLOv3 and [3] are the fastest algorithms with 10.5 and 9.7 FPS, respectively, on the proposed hardware. However, YOLO-S appears to be the best trade-off between accuracy, computing speed and memory required with a speed and a mAP improved by 37% and 16%, respectively, with respect to YOLOv3.

When handling with a small dataset, features learned from a previous task impact on model performances and, in general, the more similar the source task to the target task, the higher the performances. Specifically, using initial weights fine-tuned on domain related source images, such as in experiment 1 on DOTAv2 and VEDAI, can boost performances with respect to general features extracted in a more basic vision task such as COCO, as in experiment 2. The relative improvement ranges from a few percent for YOLO-L, YOLO-S and YOLOv3 up to 10% improved recall and 8% improved F1-score in the case of [3] and 37% better recall and 32% better F1-score for Tiny-YOLOv3. However, a single-stage fine-tuning does not always result in a performance drop of mAP and COCO metrics, especially for YOLOv3 and YOLO-L. This is in part due to the sensitivity of such metrics on class imbalance, since small absolute variations on detected instances of minority classes can lead to large fluctuations on APs, thus affecting significantly the mean scores. This is confirmed by looking at wAP, which exhibits an absolute improvement for all networks by implementing a double-stage learning and ranging from almost 1% for YOLO-L to 6% for Tiny-YOLOv3, thus proving an increased model robustness and a better generalization.

#### 3.1.2. Qualitative Evaluation

**Figure 6:**
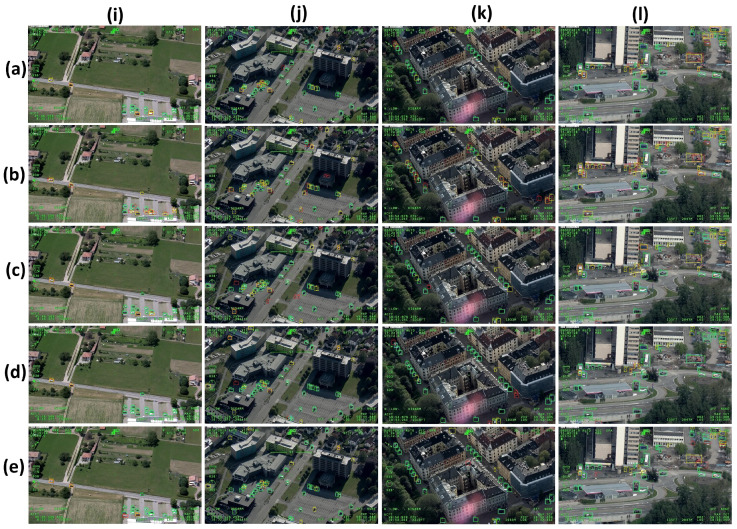
Experiment 1 on AIRES. (**a**) YOLOv3; (**b**) Tiny-YOLOv3; (**c**) [3], (**d**) YOLO-L; (**e**) YOLO-S. The following backgrounds are considered: (**i**) rural (Italy); (**j**–**l**) urban (Norway). Green (red) box denotes the true (false) positives, whereas the ground truth box is depicted as a light blue box if detection is correct and as a yellow box if a false negative occurs.

In Figure 6, we show detection results for experiment 1 on four images characterized by different background and target size. All networks outperform largely Tiny-YOLOv3. Concerning the rural scenario shown in Figure 6i, both our CNNs outperform the other networks, with two false negatives for YOLO-L, and three for YOLO-S, which have been correctly located but misclassified, up to 11 false negatives for Tiny-YOLOv3, the latter having also a very poor target localization. Similarly, false positives increase from three for YOLO-L and YOLOv3, and up to 11 for Tiny-YOLOv3.

Our networks have competitive performances also in urban scenarios, even with targets having larger size and weak contrast with ground as in Figure 6k, or size variability, partial occlusion and poor texture information as in Figure 6l. On the contrary, Tiny-YOLOv3 yields a large amount of false positives and false negatives.

### 3.2. Experiments on VEDAI Dataset

#### 3.2.1. Quantitative Comparison with State-of-the-Art Detectors

Concerning the results of experiment 1 on VEDAI dataset shown in Table 5, all architectures outperform largely Tiny-YOLOv3, which nevertheless can achieve a decent trade-off between precision and recall. Our CNNs have the most competitive results with mAP of 70.4% and 68.9% for YOLO-S and YOLO-L, respectively. Furthermore, YOLO-S outperforms the other CNNs in terms of R_ma_, whereas YOLO-L exhibits the highest balancing between precision and recall. Specifically, F1_ma_ is 83.8% for YOLO-L, 83.6% for YOLO-S and 82.2% for YOLOv3. The other detectors are instead lagging behind, with a degradation of 7% for [3] and almost 13% for Tiny-YOLOv3. Nevertheless, CNN by [3] is quite close to YOLOv3, with a mAP and wAP drop equal to 6.6% and 2.1%, respectively. YOLO-S is striking the best balance between accuracy and speed: it is up to 16% more accurate and 52% faster than YOLOv3, resulting in being only about 25% slower than Tiny-YOLOv3, and 18% slower than [3]. It is worth noticing that performances are significantly better than those in Table 4, even with smaller statistics available. Indeed, VEDAI images have been captured at the nadir and at the same altitude, unlike the AIRES dataset where point of view and acquisition height change significantly depending on camera tilt angle and helicopter altitude and making the detection consequently more challenging.

In experiment 2 on VEDAI dataset, after image resizing, the effective median target dimension spans from 13 × 13 for Car up to 37 × 37 for Plane, much smaller than the receptive fields of YOLOv3 (shown in Table 3), leaving for all CNNs a few meaningful pixels after many convolutions. As a consequence, mAP degrades by a factor 8 for Tiny-YOLOv3 and 1.6 for YOLO-S. In particular, YOLO-S exhibits still satisfactory results and outperforms all the other networks, with a mAP of 43.6% and a F1_ma_ of 73.1%. It also shows the best balancing between R_ma_ (68.8%) and P_ma_ (78.0%), which indicates that YOLO-S discriminates the artifacts from real vehicle instances well. Compared to YOLOv3, the mAP, micro F1-score, recall and precision have a relative improvement of 33.7%, 20.2%, 30.3% and 8.9%, respectively. Tiny-YOLOv3 is still significantly outperformed, also by CNN by [3], which in turn performs better than YOLOv3 for all the considered micro-average metrics and wAP.

Tiny-YOLOv3 is the fastest network processing almost 31.6 FPS. Nevertheless, YOLO-S is roughly 38% faster than YOLOV3 and only 19% slower than Tiny-YOLOv3 (14% slower than [3]). This confirms that YOLO-S is the best trade-off also on VEDAI.

#### 3.2.2. Qualitative Evaluation

**Figure 7:**
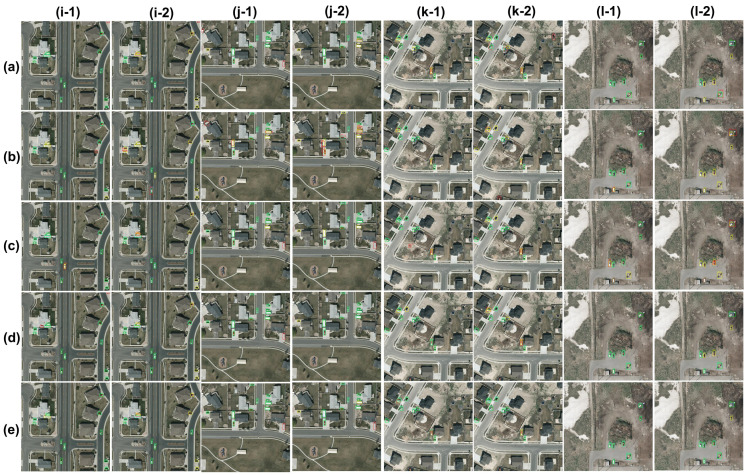
Experiments 1 (odd columns) and 2 (even columns) on VEDAI dataset. Comparison of different CNNs: (**a**) YOLOv3; (**b**) Tiny-YOLOv3; (**c**) CNN by [3]; (**d**) YOLO-L; (**e**) YOLO-S.

Figure 7 shows the detection outcomes for experiments 1 and 2 on five test images. YOLOv3, YOLO-L, YOLO-S and [3] all have remarkable performances in experiment 1 and outperform Tiny-YOLOv3. In particular, YOLO-S is the best performing network with only one missed detection in Figure 7i out of five images. YOLOv3 does not produce any false negative in Figure 7i,l. Similarly, YOLO-L detects every target in Figure 7i,j,l, and misses only a few vehicles in (k). Network by [3] also makes very few false negative and false positive samples. A generalized degradation occurs in experiment 2, especially for Tiny-YOLOv3, YOLOv3, [3] and YOLO-L, in this order. YOLO-S achieves better performances for three out of five scenarios, with YOLO-L resulting in being the best performing method for the remaining two. YOLO-S, as well as [3], is robust against vehicles with orientation. All networks outperform Tiny-YOLOv3 by a wide margin, the latter failing in any scenarios depicted in in Figure 7.

### 3.3. Experiment on SARD Dataset

#### 3.3.1. Quantitative Comparison with State-of-the-Art Detectors

Table 6 shows the accuracy and speed performances for all the aforementioned networks. Again, the proposed networks outperform the others for nearly all the detection metrics. YOLO-L has the highest AP of more than 82% and the best trade-off between precision and recall. Such values are also quite competitive with the YOLOv4 detector [4], having the same precision and a slightly better F1-score, whereas, for higher IoU thresholds, the gap is larger. It is worth noticing, however, that YOLOv4 has been trained on image resolution 512 × 512 and tested on 416 × 416, which may increase the performances a bit.

Nevertheless, YOLO-S exhibits an outstanding detection ability with an AP increase of 2.6% with respect to YOLOv3 and a higher precision for comparable recall. In addition, the highest AP_M_ for the majority category of medium-size objects proves a tighter matching between predicted and ground truth boxes for more stringent IoU thresholds. Tiny-YOLOV3 is instead by far the worst network with an AP suppressed by a factor of more than 3.5 with respect to YOLO-S. The detection speed improvement of YOLO-S is also noticeable, resulting in being 31.8% faster than YOLOv3.

#### 3.3.2. Qualitative Evaluation

**Figure 8:**
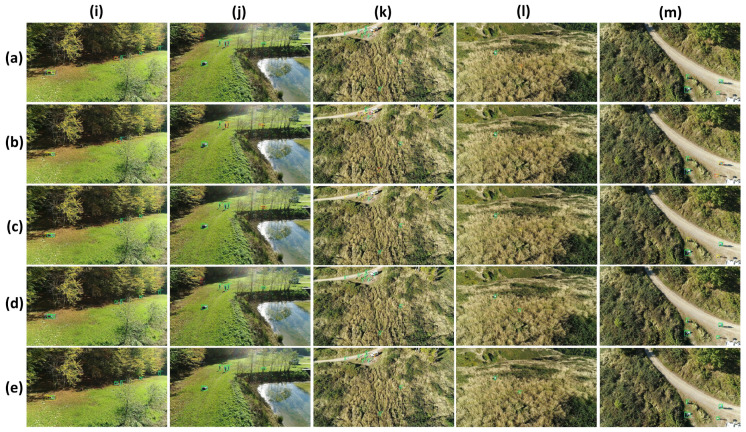
Experiment on SARD dataset. Comparison of different CNNs: (**a**) YOLOv3; (**b**) Tiny-YOLOv3; (**c**) CNN by [3]; (**d**) YOLO-L; (**e**) YOLO-S, on five different images (**i**–**m**).

In Figure 8, we compare detections on five test images. YOLO-L exhibits nearly perfect detection performances of human targets located at variable depth, having different positions such as standing, lying or crouched, as well as with a few distinctive features and poor contrast since they are hidden in high grass, as shown in Figure 8k,l, surrounded by a shady forest, as in Figure 8i, or wearing light-coloured clothes in a similar coloured dirt path as in Figure 8m. Netherless, YOLO-S also enjoys very satisfactory results with just one missed detection in Figure 8k, which has been correctly identified but poorly located, and two in Figure 8i. On the other hand, YOLOv3 tends sometimes to miss or misdetect some targets. This is because of vague features extracted after many forward convolutional layers leading to false negatives that can be seen in Figure 8i,k,l, and the coarse granularity of two output scales which may yield false positives due to poor localization of real humans, as in Figure 8i–l. The problem is of course much more severe for Tiny-YOLOv3, which is unable to detect a large amount of persons due to the absence of the YOLOv3 finest grained scale 52 × 52.

### 3.4. Summary

**Figure 9:**
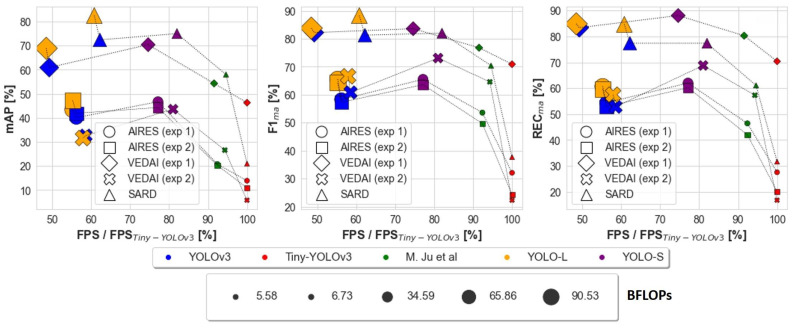
Performance summary of the different networks on AIRES, VEDAI and SARD datasets as a function of FPS. The marker size is proportional to the BFLOPs required by the network. For a better readability, FPS is normalized by Tiny-YOLOv3’s speed [3]. The model denoted as M. Ju et al. can be found in [3].

The main results are summarized in Figure 9 and can be listed as follows:.

1. By means of a sliding window method, as reported in Figure 9 for experiment 1 on AIRES dataset, YOLO-S is almost 37% faster and has a relative mAP improvement of almost 16.5% with respect to YOLOv3. Regarding experiment 1 on VEDAI dataset, YOLO-S is almost 52% faster and has a relative mAP improvement of almost 15.6% with respect to YOLOv3;
2. With respect to Tiny-YOLOv3 on AIRES dataset, YOLO-S is only about 23% slower, but has a relative F1_ma_@0.5 increase by 104.0%. On VEDAI dataset, YOLO-S is only about 25% slower than Tiny-YOLOv3, but has a relative F1_ma_@0.5 increase by 17.9%;
3. The use of pre-trained weights on a source task, such as the publicly-available DOTAv2 and VEDAI, more similar to the target domain, corresponding to AIRES dataset, leads always to a relative increase in the overall micro-average metrics @0.5 with respect to more basic preliminary features extracted on COCO dataset;
4. Inference on full-sized VEDAI images with preliminary features learned on basic COCO task leads to a F1_ma_@0.5 drop for all networks, ranging from ≈−50% for Tiny-YOLOv3 down to ≈−10% for [3] and YOLO-S, the latter having the best performance;
5. YOLO-S processes as many as 25.6 FPS on our hardware, resulting in being 38% faster than YOLOv3 and 19% slower than Tiny-YOLOv3 (14% slower than [3]);
6. YOLO-S generalizes well also to SARD dataset, with a slightly better detection accuracy than YOLOv3 and with a 32% improvement in the processing speed. Among the discussed baselines, only YOLO-L has a better average precision, but with a ≈26% slower speed and almost 2.6 times larger BFLOPs, as can be observed in Figure 9;
7. Regardless of experiments and data, YOLO-S is just from 15% to 25% slower than Tiny-YOLOv3, but even more accurate than YOLOv3 and YOLO-L;
8. YOLO-S has about one half BFLOPs of YOLOv3, quite close to SlimYOLOv3-SPP3-50 [25]. Thus, as far as power consumption is a concern like in low-power UAV applications, it is an interesting and less voluminous alternative to the by far less precise Tiny-YOLOv3.

## 4. Conclusions

We have proposed two novel YOLO-like networks designed specifically for small target detection from aerial imagery: YOLO-L and YOLO-S. YOLO-L replaces the coarse-grained output 13 × 13 of YOLOv3 with the finer-graned 104 × 104: because of a latency time close to YOLOv3, it has been presented only for benchmarking purposes. YOLO-S is instead a fast, tiny and accurate network with a single output scale 52 × 52, employing residual connection blocks to protect from a vanishing gradient issue, as well as skip connection via lateral concatenation and reshape–passthrough layer to encourage feature reuse across the network and improve consequently localization performances. In addition, we have presented an in-depth benchmarking with three baseline detectors, namely YOLOv3, Tiny-YOLOv3 and the CNN presented in [3], on our novel AIRES dataset of aerial images, as well as on VEDAI and SARD datasets.

Simulations have been conducted either by implementing a detection pipeline based on a sliding window approach and an NMS post-processing to handle full-sized images or by means of a single-shot inference on the whole images. The objective of this work was to propose a model, YOLO-S that is small and portable, even smaller than Tiny-YOLOv3, but do not sacrifice in accuracy with respect to larger models such as YOLOv3. In fact, Tiny-YOLOv3 is small and fast, but it is quite inaccurate for small object detection. On the other hand, YOLO-S is as much as small as Tiny-YOLOv3 but not so fast, being the architecture more complex. As a result, YOLO-S is more accurate and faster than the large model YOLOv3, but it is not so fast as Tiny-YOLOv3. We sacrifice speed for accuracy with respect to Tiny-YOLOv3, still obtaining a very small model that is more accurate and faster then the larger ones.

In summary, YOLO-S can really meet accuracy and real-time detection requirements and is consequently a promising candidate for integration on low-power GPU-less systems. The next steps include the investigation of structured pruning and data quantization techniques [47] to reduce further the computational complexity and make the network even smaller and faster.

## Figures and Tables

**Table 1:** Number of GT objects in the global, training and test sets for each class for AIRES, VEDAI and SARD datasets. DOTAv2 is not shown since it is used only for training.

		AIRES			VEDAI			SARD	
Class	Global	Train	Test	Global	Train	Test	Global	Train	Test
Van	2012	1437	575	498	350	148	-	-	-
Truck	755	534	221	307	221	86	-	-	-
Car	10,518	7520	2998	2332	1676	656	-	-	-
Motorbike	83	61	22	-	-	-	-	-	-
Person	990	792	198	-	-	-	6525	5220	1305
Other	123	89	34	204	157	47	-	-	-
Boat	498	349	149	171	120	51	-	-	-
Bus	268	194	74	-	-	-	-	-	-
Tractor	-	-	-	190	137	53	-	-	-
Plane	-	-	-	48	36	12	-	-	-
# of GTs	15,247	10,976	4271	3750	2697	1053	6525	5220	1305
# of Images	1275	898	377	1246	892	354	1980	1601	379

**Table 2:** The architecture of the proposed networks YOLO-L (**left**) and YOLO-S (**right**). F: number of convolutional filters; S/S: kernel size/stride; CS: cumulative stride; RF: receptive field; R: residual block. In the table, C=80 classes have been assumed (dataset COCO).

				YOLO-L							YOLO-S			
**#**	**Type**	**F**	**S/S**	**Input**	**Output**	**CS**	**RF**	**Type**	**F**	**S/S**	**Input**	**Output**	**CS**	**RF**
0	Conv	32	3/1	416 × 416 × 3	416 × 416 × 32	1	3	Conv	32	3/1	416 × 416 × 3	416 × 416 × 32	1	3
1	Conv	64	3/2	416 × 416 × 32	208 × 208 × 64	2	5	Conv	64	3/2	416 × 416 × 32	208 × 208 × 64	2	5
2	Conv (R1)	32	1/1	208 × 208 × 64	208 × 208 × 32	2	5	Conv (R1)	32	1/1	208 × 208 × 64	208 × 208 × 32	2	5
3	Conv (R1)	64	3/1	208 × 208 × 32	208 × 208 × 64	2	9	Conv (R1)	64	3/1	208 × 208 × 32	208 × 208 × 64	2	9
4	Conv	128	3/2	208 × 208 × 64	104 × 104 × 128	4	13	Conv	128	3/2	208 × 208 × 64	104 × 104 × 128	4	13
5	Conv (R1)	64	1/1	104 × 104 × 128	104 × 104 × 64	4	13	Conv (R1)	64	1/1	104 × 104 × 128	104 × 104 × 64	4	13
6	Conv (R1)	128	3/1	104 × 104 × 64	104 × 104 × 128	4	21	Conv (R1)	128	3/1	104 × 104 × 64	104 × 104 × 128	4	21
7	Conv (R2)	64	1/1	104 × 104 × 128	104 × 104 × 64	4	21	Conv (R2)	64	1/1	104 × 104 × 128	104 × 104 × 64	4	21
8	Conv (R2)	128	3/1	104 × 104 × 64	104 × 104 × 128	4	29	Conv (R2)	128	3/1	104 × 104 × 64	104 × 104 × 128	4	29
9	Conv	256	3/2	104 × 104 × 128	52 × 52 × 256	8	37	Conv	256	3/2	104 × 104 × 128	52 × 52 × 256	8	37
10	Conv (R1)	128	1/1	52 × 52 × 256	52 × 52 × 128	8	37	Conv (R1)	128	1/1	52 × 52 × 256	52 × 52 × 128	8	37
11	Conv (R1)	256	3/1	52 × 52 × 128	52 × 52 × 256	8	53	Conv (R1)	256	3/1	52 × 52 × 128	52 × 52 × 256	8	53
12	Conv (R2)	128	1/1	52 × 52 × 256	52 × 52 × 128	8	53	Conv (R2)	128	1/1	52 × 52 × 256	52 × 52 × 128	8	53
13	Conv (R2)	256	3/1	52 × 52 × 128	52 × 52 × 256	8	69	Conv (R2)	256	3/1	52 × 52 × 128	52 × 52 × 256	8	69
14	Conv (R3)	128	1/1	52 × 52 × 256	52 × 52 × 128	8	69	Conv	512	3/2	52 × 52 × 256	26 × 26 × 512	16	85
15	Conv (R3)	256	3/1	52 × 52 × 128	52 × 52 × 256	8	85	Conv (R1)	256	1/1	26 × 26 × 512	26 × 26 × 256	16	85
16	Conv (R4)	128	1/1	52 × 52 × 256	52 × 52 × 128	8	85	Conv (R1)	512	3/1	26 × 26 × 256	26 × 26 × 512	16	117
17	Conv (R4)	256	3/1	52 × 52 × 128	52 × 52 × 256	8	101	Conv (R2)	256	1/1	26 × 26 × 512	26 × 26 × 256	16	117
18	Conv (R5)	128	1/1	52 × 52 × 256	52 × 52 × 128	8	101	Conv (R2)	512	3/1	26 × 26 × 256	26 × 26 × 512	16	149
19	Conv (R5)	256	3/1	52 × 52 × 128	52 × 52 × 256	8	117	Conv	128	1/1	26 × 26 × 512	26 × 26 × 128	16	149
20	Conv (R6)	128	1/1	52 × 52 × 256	52 × 52 × 128	8	117	Upsample		2/1	26 × 26 × 128	52 × 52 × 128	16	149
21	Conv (R6)	256	3/1	52 × 52 × 128	52 × 52 × 256	8	133	Route 8						
22	Conv (R7)	128	1/1	52 × 52 × 256	52 × 52 × 128	8	133	Reshape			104 × 104 × 128	52 × 52 × 512	4	29
23	Conv (R7)	256	3/1	52 × 52 × 128	52 × 52 × 256	8	149	Route 22,20,13						
24	Conv (R8)	128	1/1	52 × 52 × 256	52 × 52 × 128	8	149	Conv	256	1/1	52 × 52 × 896	52 × 52 × 256	8	69
25	Conv (R8)	256	3/1	52 × 52 × 128	52 × 52 × 256	8	165	Conv	512	3/1	52 × 52 × 256	52 × 52 × 512	8	85
26	Conv	512	3/2	52 × 52 × 256	26 × 26 × 512	16	181	Conv	256	1/1	52 × 52 × 512	52 × 52 × 256	8	85
27	Conv (R1)	256	1/1	26 × 26 × 512	26 × 26 × 256	16	181	Conv	512	3/1	52 × 52 × 256	52 × 52 × 512	8	101
28	Conv (R1)	512	3/1	26 × 26 × 256	26 × 26 × 512	16	213	Conv	255	1/1	52 × 52 × 512	52 × 52 × 255	8	101
29	Conv (R2)	256	1/1	26 × 26 × 512	26 × 26 × 256	16	213	Yolo						
30	Conv (R2)	512	3/1	26 × 26 × 256	26 × 26 × 512	16	245							
31	Conv (R3)	256	1/1	26 × 26 × 512	26 × 26 × 256	16	245							
32	Conv (R3)	512	3/1	26 × 26 × 256	26 × 26 × 512	16	277							
33	Conv (R4)	256	1/1	26 × 26 × 512	26 × 26 × 256	16	277							
34	Conv (R4)	512	3/1	26 × 26 × 256	26 × 26 × 512	16	309							
35	Conv (R5)	256	1/1	26 × 26 × 512	26 × 26 × 256	16	309							
36	Conv (R5)	512	3/1	26 × 26 × 256	26 × 26 × 512	16	341							
37	Conv (R6)	256	1/1	26 × 26 × 512	26 × 26 × 256	16	341							
38	Conv (R6)	512	3/1	26 × 26 × 256	26 × 26 × 512	16	373							
39	Conv (R7)	256	1/1	26 × 26 × 512	26 × 26 × 256	16	373							
40	Conv (R7)	512	3/1	26 × 26 × 256	26 × 26 × 512	16	405							
41	Conv (R8)	256	1/1	26 × 26 × 512	26 × 26 × 256	16	405							
42	Conv (R8)	512	3/1	26 × 26 × 256	26 × 26 × 512	16	437							
43	Conv	256	1/1	26 × 26 × 512	26 × 26 × 256	16	437							
44	Conv	512	3/1	26 × 26 × 256	26 × 26 × 512	16	469							
45	Conv	256	1/1	26 × 26 × 512	26 × 26 × 256	16	469							
46	Conv	512	3/1	26 × 26 × 256	26 × 26 × 512	16	501							
47	Conv	256	1/1	26 × 26 × 512	26 × 26 × 256	16	501							
48	Conv	512	3/1	26 × 26 × 256	26 × 26 × 512	16	533							
49	Conv	255	1/1	26 × 26 × 512	26 × 26 × 255	16	533							
50	Yolo													
51	Route 47													
52	Conv	128	1/1	26 × 26 × 256	26 × 26 × 128	16	501							
53	Upsample		2/1	26 × 26 × 128	52 × 52 × 128	16	501							
54	Route 53,25													
55	Conv	256	1/1	52 × 52 × 384	52 × 52 × 256	8	165							
56	Conv	512	3/1	52 × 52 × 256	52 × 52 × 512	8	181							
57	Conv	256	1/1	52 × 52 × 512	52 × 52 × 256	8	181							
58	Conv	512	3/1	52 × 52 × 256	52 × 52 × 512	8	197							
59	Conv	256	1/1	52 × 52 × 512	52 × 52 × 256	8	197							
60	Conv	512	3/1	52 × 52 × 256	52 × 52 × 512	8	213							
61	Conv	255	1/1	52 × 52 × 512	52 × 52 × 255	8	213							
62	Yolo													
63	Route 59													
64	Conv	128	1/1	52 × 52 × 256	52 × 52 × 128	8	197							
65	Upsample		2/1	52 × 52 × 128	104 × 104 × 128	8	197							
66	Route 65,8													
67	Conv	128	1/1	104 × 104 × 256	104 × 104 × 128	4	29							
68	Conv	256	3/1	104 × 104 × 128	104 × 104 × 256	4	37							
69	Conv	128	1/1	104 × 104 × 256	104 × 104 × 128	4	37							
70	Conv	256	3/1	104 × 104 × 128	104 × 104 × 256	4	45							
71	Conv	128	1/1	104 × 104 × 256	104 × 104 × 128	4	45							
72	Conv	256	3/1	104 × 104 × 128	104 × 104 × 256	4	53							
73	Conv	255	1/1	104 × 104 × 256	104 × 104 × 255	4	53							
74	Yolo													

**Table 3:** Number of parameters, volume, BFLOPs, output scales (OS), receptive field (RF) and cumulative stride (CS) for each network. The lightest (2nd lightest) model is highlighted in black (green). The two CNNs having smaller BFLOPs are also highlighted.

Net	Par [M]	Vol [MB]	BFLOPs	OS	RF	CS
				13	917	32
YOLOv3	61.6	242.9	65.86	26	533	16
				52	213	8
Tiny-YOLOv3	8.7	34.7	**5.58**	13	318	32
				26	110	16
Ref. [3]	**0.7**	**3.4**	**6.73**	52	133	8
				26	533	16
YOLO-L	23.8	94.9	90.53	52	213	8
				104	53	4
YOLO-S	**7.8**	**31.9**	34.59	52	101	8

**Table 4:** Comparative results of experiments 1 and 2 on AIRES dataset. The 1st (2nd) best score is highlighted in black (green) for each experiment. The metric shown for each class is AP[%].

	YOLOv3		Tiny-YOLOv3		Reference [3]		YOLO-L		YOLO-S	
Metric [%]	Exp. 1	Exp. 2	Exp. 1	Exp. 2	Exp. 1	Exp. 2	Exp. 1	Exp. 2	Exp. 1	Exp. 2
Van	33.1	33.5	9.1	4.4	18.4	19.4	**36.6**	**38.2**	**44.0**	**39.2**
Truck	47.1	47.4	17.5	12.3	17.0	18.6	**47.3**	**50.3**	**50.5**	**48.1**
Car	47.8	44.9	19.1	12.2	46.5	40.6	**57.3**	**54.8**	**58.7**	**57.0**
Motorbike	**20.4**	**32.6**	11.4	9.1	0.0	0.0	**26.6**	**23.9**	13.6	20.5
Person	35.5	**44.3**	6.4	6.0	15.2	12.6	**47.6**	**47.3**	**42.4**	41.8
Other	**39.7**	**37.6**	6.5	3.8	0.0	5.9	20.3	**40.5**	**39.9**	32.1
Boat	39.5	34.5	13.5	10.1	44.8	34.6	**52.5**	**60.3**	**65.9**	**59.0**
Bus	**57.7**	**60.1**	28.2	29.6	24.3	31.0	**58.9**	**63.0**	**58.9**	56.8
mAP	40.1	41.9	13.9	10.9	20.8	20.3	**43.4**	**47.3**	**46.7**	**44.3**
wAP	44.9	43.2	16.9	11.0	38.7	34.5	**52.9**	**52.0**	**55.4**	**53.1**
AP_S_	5.5	6.8	0.8	0.4	3.8	2.8	**11.9**	**11.5**	**10.4**	**9.4**
AP_M_	17.5	17.4	5.1	3.1	11.3	11.4	**21.6**	**22.4**	**25.4**	**23.7**
AP_L_	**28.1**	**31.7**	11.1	10.5	10.9	10.2	**29.3**	**31.0**	22.1	22.8
R_ma_	54.4	52.9	27.5	20.1	46.4	42.0	**60.7**	**59.7**	**62.0**	**60.2**
P_ma_	62.9	62.6	38.6	30.9	63.4	60.9	**71.4**	**69.6**	**69.5**	**67.3**
F1_ma_	58.3	57.3	32.1	24.3	53.6	49.7	**65.6**	**64.3**	**65.5**	**63.6**
FPS	5.9	5.9	**10.5**	**10.5**	**9.7**	**9.7**	5.8	5.8	8.1	8.1

**Table 5:** Comparative results of experiments 1 and 2 on VEDAI dataset. The 1st (2nd) best score is highlighted in black (green) for each experiment. The metric shown for each class is AP[%].

Metric [%]	YOLOv3		Tiny-YOLOv3		Reference [3]		YOLO-L		YOLO-S	
	Exp. 1	Exp. 2	Exp. 1	Exp. 2	Exp. 1	Exp. 2	Exp. 1	Exp. 2	Exp. 1	Exp. 2
Van	**62.6**	31.4	38.7	0.4	54.2	27.1	59.4	**38.2**	**70.7**	**44.5**
Truck	51.1	13.9	35.4	1.4	40.3	12.4	**60.3**	**30.1**	**65.5**	**32.6**
Car	91.3	59.2	80.8	10.6	**93.1**	**71.4**	91.5	66.3	**95.5**	**80.6**
Tractor	39.0	4.3	21.6	0.5	42.3	**4.5**	**70.6**	3.5	**63.8**	**27.3**
Other	30.7	15.2	33.6	0.7	25.0	9.6	**39.4**	**16.0**	**47.9**	**19.9**
Plane	86.7	**96.1**	**89.9**	26.4	81.1	57.3	**98.7**	62.9	75.0	**74.2**
Boat	**65.2**	**7.9**	23.9	1.0	44.5	3.9	62.5	4.0	**74.2**	**25.9**
mAP	60.9	**32.6**	46.3	5.8	54.3	26.6	**68.9**	31.6	**70.4**	**43.6**
wAP	77.3	44.8	63.4	7.2	75.2	50.8	**79.7**	**50.9**	**84.6**	**63.5**
AP_S_	**24.2**	6.7	9.4	0.9	16.4	6.1	22.8	**8.7**	**30.8**	**9.4**
AP_M_	35.4	10.0	25.0	1.4	31.4	12.1	**39.6**	**13.5**	**41.8**	**22.2**
AP_L_	30.3	**23.6**	23.4	6.9	23.9	13.9	**39.0**	**30.6**	**31.9**	22.8
R_ma_	83.4	52.8	70.4	16.7	80.3	57.3	**84.9**	**57.5**	**88.1**	**68.8**
P_ma_	**81.1**	71.6	71.5	33.5	73.6	74.2	**82.7**	**78.9**	79.6	**78.0**
F1_ma_	82.2	60.8	70.9	22.3	76.8	64.6	**83.8**	**66.6**	**83.6**	**73.1**
FPS	6.4	18.5	**13.0**	**31.6**	**11.9**	**29.8**	6.3	18.3	9.7	25.6

**Table 6:** Experiment on SARD dataset. The 1st (2nd) best score is highlighted in black (green). Results from [4] for YOLOv4 are shown on the right.

Metric [%]	YOLOv3	Tiny-Yv3	Reference [3]	YOLO-L	YOLO-S	YOLOv4 [4]
AP	72.4	21.0	58.0	**82.5**	**75.0**	88.0
AP_S_	**13.4**	1.6	4.4	**18.9**	11.2	26.0
AP_M_	32.9	5.6	28.2	**41.5**	**42.7**	59.0
AP_L_	45.3	23.7	47.0	**55.6**	**61.2**	75.0
REC_ma_	**77.4**	31.6	61.1	**84.7**	**77.4**	89.8
PREC_ma_	85.6	46.9	82.9	**92.2**	**87.1**	92.7
F1_ma_	81.3	37.7	70.4	**88.3**	**82.0**	91.2
FPS	17.3	**27.8**	**26.3**	16.9	22.8	NA

## Data Availability

Not applicable.

## Abbreviations

The following abbreviations are used in this manuscript:

YOLO	You Only Look Once
AIRES	cAr detectIon fRom hElicopter imageS
VEDAI	Vehicle Detection in Aerial Imagery
DOTA	Dataset for Object deTection in aerial images
COCO	Common Objects in Context
SARD	Search and Rescue Image Dataset for Person Detection
FLOPs	FLoating point Operations Per Second
BFLOPs	Billion FLoating point Operations Per Second
UAV	Unmanned Aerial Vehicle
CNN	Convolutional Neural Network
GPU	Graphics Processing Unit
GT	Ground Truth
FHD	Full High-Definition
FPN	Feature Pyramid Network
NMS	Non Maximal Suppression
mAP	mean Average Precision
AP	Average Precision
wAP	weighted Average Precision
IoU	Intersection over Union
REC	recall
PREC	precision
FPS	Frames Per Second
